# Technology innovation to reduce health inequality in skin diagnosis and to improve patient outcomes for people of color: a thematic literature review and future research agenda

**DOI:** 10.3389/frai.2024.1394386

**Published:** 2024-06-13

**Authors:** Nazma Khatun, Gabriella Spinelli, Federico Colecchia

**Affiliations:** College of Engineering, Design and Physical Science, Brunel Design School, Brunel University London, Uxbridge, United Kingdom

**Keywords:** dermatology, artificial intelligence, skin of color, people of color, ethnic minorities, data augmentation, health inequalities

## Abstract

The health inequalities experienced by ethnic minorities have been a persistent and global phenomenon. The diagnosis of different types of skin conditions, e.g., melanoma, among people of color is one of such health domains where misdiagnosis can take place, potentially leading to life-threatening consequences. Although Caucasians are more likely to be diagnosed with melanoma, African Americans are four times more likely to present stage IV melanoma due to delayed diagnosis. It is essential to recognize that additional factors such as socioeconomic status and limited access to healthcare services can be contributing factors. African Americans are also 1.5 times more likely to die from melanoma than Caucasians, with 5-year survival rates for African Americans significantly lower than for Caucasians (72.2% vs. 89.6%). This is a complex problem compounded by several factors: ill-prepared medical practitioners, lack of awareness of melanoma and other skin conditions among people of colour, lack of information and medical resources for practitioners’ continuous development, under-representation of people of colour in research, POC being a notoriously hard to reach group, and ‘whitewashed’ medical school curricula. Whilst digital technology can bring new hope for the reduction of health inequality, the deployment of artificial intelligence in healthcare carries risks that may amplify the health disparities experienced by people of color, whilst digital technology may provide a false sense of participation. For instance, Derm Assist, a skin diagnosis phone application which is under development, has already been criticized for relying on data from a limited number of people of color. This paper focuses on understanding the problem of misdiagnosing skin conditions in people of color and exploring the progress and innovations that have been experimented with, to pave the way to the possible application of big data analytics, artificial intelligence, and user-centred technology to reduce health inequalities among people of color.

## 1 Introduction

Healthcare inequalities have been persistent throughout healthcare globally (Stuart and Soulsby, 2011). These imbalances are present in healthcare access, treatments, and outcomes among minority communities (WHO, 2018) and can lead to detrimental health consequences. Disparity in health outcomes can be based on several factors such as gender, age, ethnicity, access to support and care services, and familiarity with digital technology. Digital technology, including artificial intelligence (AI), has been implemented into several areas of healthcare to combat inequalities. Despite targeted approaches, challenges associated with resource constraints and unintentional biases pose threats to successful execution and development, predominantly for people of color (POC).

Studies have illustrated the use of AI within dermatological settings for skin diagnostics of lesions including melanoma. Melanoma is a common type of skin cancer that originates from melanocyte skin cells (Cancer Council, 2023). Recognising signs of melanoma is crucial for early detection: lesions often appear as moles undergoing changes in color, growth patterns, shape irregularities, or being elevated and itchy (Cancer Council, 2023). Unfortunately, POC are at a greater disadvantage in melanoma mortality rates for reasons including late diagnosis or incorrect treatment (Mahendraraj et al., 2017), the integration of AI could address these issues by benefiting both healthcare workers and POC, considering internal medicine and physician trainees were less likely to refer POC to specialists for further management, with only 25% of trainees referring a drug rash for POC compared to 40% for Caucasian patients (Hutchison et al., 2023).

This paper explores the role of digital technology and AI to reduce health inequality, while also evaluating the benefits and challenges of AI adoption. The use of AI in diagnosing skin conditions, especially among POC, has the potential to magnify existing health inequalities for POC. This paper is concerned with diagnostic accuracy, equity in healthcare, potential biases in the technology, and the use of appropriate terminology to enable a more considerable adoption of digital health technologies.

## 2 Methodology

**Figure 1:**
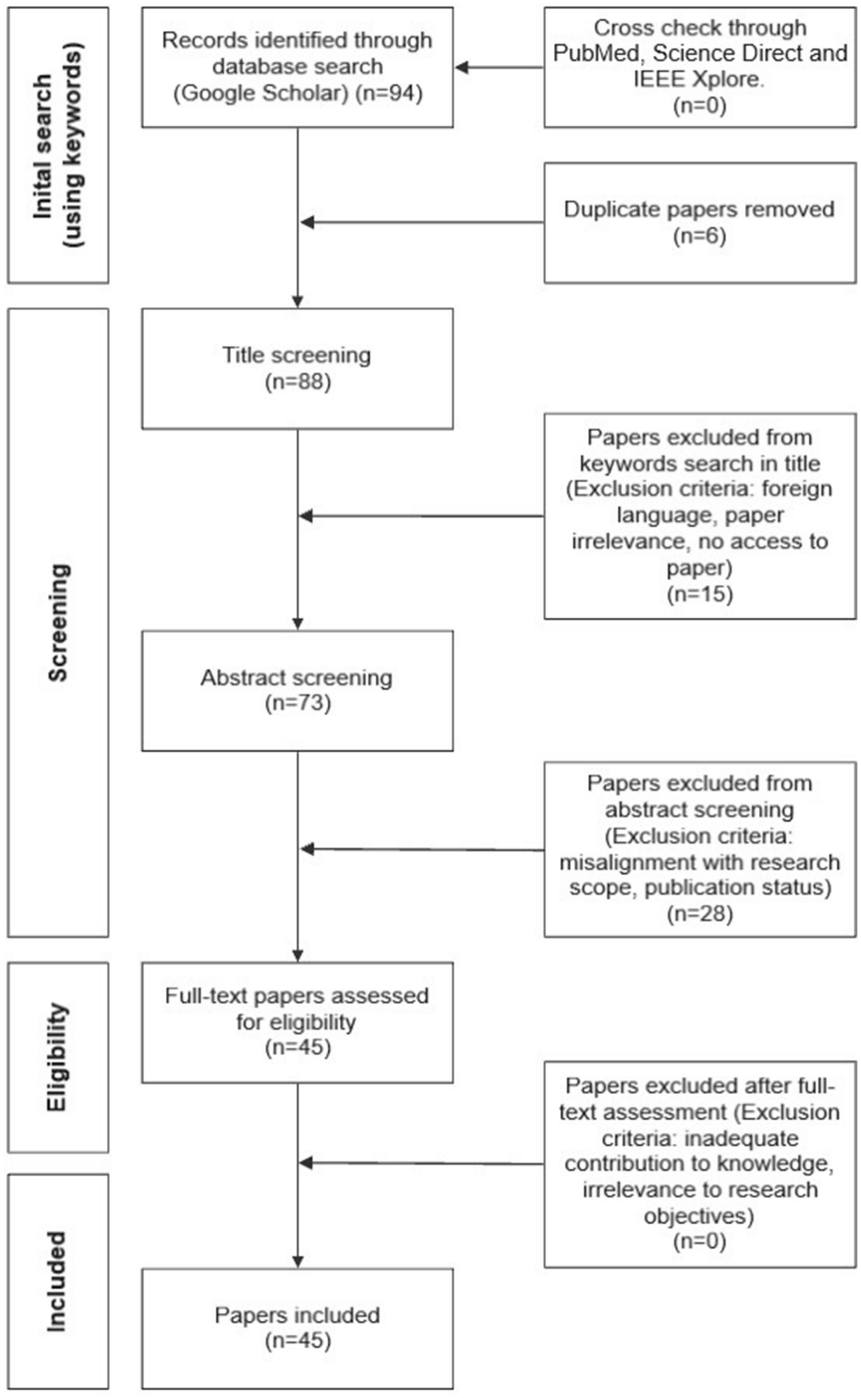
Flowchart of the selection process to identify target papers.

For this literature review, an opportunistic search was carried out through Google and Google Scholar. The interconnection of health inequality, dermatology, and AI was investigated in several fields of research including engineering, computing, medicine, and healthcare by selecting relevant keywords. Only published academic literature and grey literature from reputable sources (e.g., American Journal of Clinical Dermatology and International Journal of Equity in Health) were selected. A total of 94 relevant papers were shortlisted based on the matching between keywords and the papers’ title. A further selection took place following the review of the abstracts. This led to 45 publications (42 academic papers and 3 conferences) that were determined appropriate and relevant for this review. Other research databases, including PubMed, Science Direct and IEEE Xplore, have also been searched using the same selection criteria to ensure all recent, and key literature has been identified and included. From this cross-check, no new additional papers have been identified. Geographical locations or date of publication were not restricting factors. This was to ensure that all potential AI advancements in skin lesion recognition and approaches to mitigating health inequality were explored. Papers were selected regardless of whether the studies had POC representation; if a paper had information on skin tone or ethnicity, it was considered. This was to ensure a comprehensive understanding of the problem was identified, to remove chances of biases, and for a clear and transparent comparative analysis of skin color representation within AI. Literature not written in English was not considered to avoid the chances of misinterpreting any findings. Biases have also been mitigated by defining and using consistently certain keywords, which collectively establish the objective criteria for papers’ selection at the title screening level (see Figure 1). This method ensured that the selection process was based on specific, predefined criteria rather than subjective judgment, resulting in reduced chances of potential bias. Papers were screened by all three authors, and any discrepancies were resolved through discussions. Taking this approach allowed for a transparent review process. Figure 1 shows a flowchart of the selection process to identify target papers.

The following combination of keywords was used to identify relevant papers: ‘artificial intelligence within dermatology,” ‘people of color and skin diagnosis accuracy in artificial intelligence’, ‘clinical pathway and artificial intelligence use’, ‘skin diagnosis tools for people of color’, ‘AI skin diagnosis in people of color’, ‘Artificial intelligence use within healthcare’, ‘digital technology to reduce healthcare inequalities’, ‘artificial intelligence’, ‘overfitting in artificial intelligence and skin diagnosis’, ‘data augmentation in artificial intelligence and skin diagnosis’, ‘image selection for artificial intelligence and skin diagnosis’, ‘people of color representation within artificial intelligence’, ‘artificial intelligence vs. experts diagnosis accuracy of skin disease’, ‘health inequality’, and ‘language barriers’. The search for relevant literature stopped upon reaching saturation, where no additional literature matching the keywords could be found. The search end date was March 2024, to ensure the most recent publications were considered.

The initial search on Google Scholar was undertaken using the keywords previously listed. This search identified 94 papers. A comparison with searches on other scientific databases did not identify additional papers. The initial search identified 6 duplicate papers that were removed from the set prior to reaching title screening. 88 papers reached the title screening level and 15 were excluded at this stage. 73 papers reached the abstract screening level and 28 were excluded. 45 were considered eligible for full review. At this stage, no papers were excluded. The final set of papers considered in this review was 45.

## 3 Results

### 3.1 Digital technologies to reduce health inequality

Digital technology plays an important role in addressing and presenting opportunities to overcome several barriers within health inequality. Deployment of technology can be through virtual health services, telemedicine consultations, or educational initiatives. Technologies as such benefit marginalized communities that may be constrained by geographical locations, financial situations, or inadequacies in equal access to healthcare resources and services for all (Table 1).

**Table 1:** Literature sourced organized by theme. Some references fit into multiple categories due to their overlapping relevance.

**Category**	**Reference**	**No. of papers**
Understanding of the healthcare system, dermatology, and skin conditions	DermNet (2012), Eedy (2015), Mahendraraj et al. (2017), Chuchu et al., (2018), Goddu et al. (2018), Johnson et al. (2022), Al-Janabi et al. (2023), Cancer Council (2023), Heldreth et al. (2024), and Department of Health and Social Care, (2024)	8
Exploration of health inequality faced by POC	Hutchison et al. (2023), Kelly and Haidet (2007), Kenison et al. (2017), Stuart and Soulsby (2011), WHO (2018), Lester et al. (2019), Chauhan et al. (2020) and Raney et al. (2021)	8
Solutions to address skin diagnosis inequality faced by POC	Mukwende (2020), St. George’s University (2020), Smith (2021), NHS (2021a), and NHS (2022)	5
Understanding of AI	Mitrani (2019) and Du-Harpur et al. (2020)	2
Current uses of AI in healthcare and skin diagnosis	Hakim (2023), Healthy.io (2024), Lacobucci, 2023, NHS (2021a), Obermeyer et al. (2019), Schakermann et al. (2024), Shore et al. (2019) and While (2023)	8
Understanding of AI in Skin diagnosis	Nasr-Esfahani et al. (2016), Aggarwal (2019), Brinker et al. (2019), Mitrani (2019), Khosla and Saini (2020), and Nahm (2022)	6
Understanding of data augmentation in AI	Perez et al. (2018), Aggarwal (2019), Chlap et al. (2021), Wen et al., 2022, Abayomi-Alli et al. (2021), Rezk et al. (2022) and Saeed et al. (2023)	7
Understanding of image selection in AI	Ribeiro et al. (2016), Koziarski and Cyganek, 2018, Aggarwal (2019), Brinker et al. (2019), Hogarty et al. (2019), Winkler et al., 2019 and Liopyris et al. (2022)	7
AI performance compared to Dermatologists	Brinker et al. (2019), Han et al. (2020), Philips et al. (2020)	3
AI performance with POC representation	Chen et al. (2016), Jinnai et al. (2020), Liu et al. (2020), and Liu and Primiero (2023)	4
Data augmentation to increase POC data	Aggarwal (2019) and Abhari and Ashok (2023)	2
Assessment of Skin Image Search	Zaar et al. (2020), Kamulegeya et al. (2023), and (2021)	3
Google AI development	Bui and Liu (2021) and Liu et al. (2020)	2

The Core20PLUS5 is a national NHS strategy to reduce health inequalities on a system and national level. The approach identifies target populations among adults, young people and children, and clinical areas that need improvement (NHS, 2021a). Core20PLUS5 has three components: Core20 refers to the 20% of the most deprived national population, identified by the Index of Multiple Deprivation (IMD), PLUS relates to individuals including ethnic minorities or groups defined by the Equality Act 2010, and 5 stands for the five clinical areas which need improvement including severe mental illness or early diagnosis of cancer (NHS, 2021a). The strategy provides platforms, builds networks, and creates opportunities for sharing best practices. The targeted approach of Core20PLUS5 demonstrates clinical priority areas being addressed to attain health equality and inclusivity. However, the success of the recently developed approach relies on robust monitoring and evaluation to ensure the program is continuously relevant and appropriate.

The clinical pathway within the UK and globally has shown that a choice of language matters when describing medical conditions (Chauhan et al., 2020; NHS, 2022). This can be for reasons including the reoccurrence of negative biases (Goddu et al., 2018; Raney et al., 2021), difficulty in understanding the choice of terminology (Kelly and Haidet, 2007; Kenison et al., 2017) or irrelevancy for minority groups through descriptions of medical conditions and images (NHS, 2022). The issue of language is evident within the NHS, particularly in the implementation of the comprehensive digital tool, Health A-Z (NHS, 2022). Health A-Z is designed to provide information on conditions, symptoms, and treatments for the public; however, at times, it fails to provide relevant symptom descriptions for all groups of people. When addressing skin conditions, the language used tends to focus on physical appearances and is often tailored to Caucasian skin types. While beneficial for some, it often leads to confusion among minority groups including POC or the visually impaired. Descriptions like “becoming pale” or “lips turning blue” may be relevant for Caucasians but may be challenging for minority groups to interpret (St. George’s University, 2020). Smith (2021), a content designer for the NHS website, revealed patients want inclusive language such as “there are approximately ten spots that vary in size from about 1 mm to 1 cm, some spots are close together” to describe chickenpox which offers a neutral description that is independent from color reference. The implementation of a more neutral and objective language is underway, but the lack of medical sources detailing symptoms on Brown and Black skin poses a challenge to accurately describe how symptoms appear on diverse skin tones, slowing down the creation of inclusive material and the adoption of a neutral language.

Inadequate resources and knowledge for skin lesion diagnosis in POC is a persistent issue. Malone Mukwende, a medical student, developed Mind the Gap (Mukwende, 2020) after identifying a gap in the representation of POC in medical textbooks. Mind the Gap is a free online photographic repository with and without supporting text descriptions of various skin conditions with Fitzpatrick scale (FST) V and VI (DermNet, 2012). This tool is used worldwide in educational and professional settings (St. George’s University, 2020) and relies on the public information sharing of skin conditions. The initiative addresses the representation gap and enhances global accessibility to a valuable resource, but the reliance on external contribution can stagnate the growth of the digital tool. There is also a risk of individuals self-misdiagnosing skin conditions if there is a lack of professional follow-up.

### 3.2 Artificial intelligence to reduce health inequality

AI describes the ability of machines to learn, communicate, reason, conduct different tasks simultaneously, or operate independently in different scenarios similarly to humans (Hogarty et al., 2019; Du-Harpur et al., 2020). Within the realm of AI, machine learning can be supervised, semi-supervised or unsupervised (Hogarty et al., 2019), depending on the level of human intervention in correcting and directing the machine learning process.

Numerous instances of AI implementations within the clinical process have demonstrated promising outcomes in addressing health inequalities but have drawn attention to underlying issues. Examples of AI integration are Healthy.io and mobile applications such as Mindful Kidney (Healthy.io, 2024). The self-testing urine kit produces real-time clinical results through colorimetric analysis, computer vision, AI, and a smartphone camera that transforms into a clinical-grade medical device (NHS, 2021b; Healthy.io, 2024). This AI-powered digital technology reduces health inequality through accessibility to remote testing which may be challenging for some due to cost, transportation, or geographical locations. Findings show that patients favor the use of Healthy.io over taking a urine sample at their GP, possibly due to the comfort of their home and the ability to conduct the test at a convenient time (Shore et al., 2019). Considering user requirements can contribute to the success of AI integration; however ethical concerns have risen from a pilot study at a GP based in Oxford, where patient data were shared with a third party. This consequently led to the GP withdrawing from the study (Lacobucci, 2023) because the study became perceived as one with high risks for patients’ privacy.

The Virtual AI Ward treating remote patients hosted by the NHS Croydon Primary Care Trust demonstrated the potential of AI. All users reported positive outcomes, especially regarding the ease of learning and understanding of the provided medical kits; the overall experience led to an improvement in participants’ quality of life (Hakim, 2023). Success of the Virtual AI Ward was attributed to being run by community services with pathways to emergency treatment, when needed, upskilled staff, knowing when to choose continuous monitoring over spot monitoring, and having access to a cross-system multi-disciplinary team (Hakim, 2023; While, 2023). Challenges within the NHS including underfunding, understaffing, and overworked staff (Johnson et al., 2022; Al-Janabi et al., 2023), could adversely impact the success rates of implementing Virtual Wards across the NHS.

The US-based study by Obermeyer et al. (2019) explores the integration of AI into a medical system used within hospitals that raised ethical concerns. The AI program aims to predict complex health needs for the purpose of developing an intervention that manages those in need (Obermeyer et al., 2019). Patients are enrolled in the AI system through their insurance program if their risk score falls above the 97th percentile. The metadata gathered for the AI program includes demographic, insurance type, diagnoses, medications, and detailed costs, but specifically excludes race. Obermeyer et al. (2019) suggest that the algorithm’s prediction on health needs is based on costing. Black and Caucasian patients have roughly the same costs per year, with Black patients generating an average of $1,801 less than Caucasians annually, despite having 26.3% more ongoing health issues. This suggests that the AI program failed to highlight health needs by predicting an equal level of risk for both groups. Identifying this, Obermeyer et al. (2019) adjusted the labels used within the algorithm, inevitably showing an increase in the percentage of additional help received by Black patients from 17.7 to 46.5%. This study is a distinct example of biases and ethical concerns that arise inversely through label choices, affecting predictive performance and creating racial biases, and exhibits why AI needs close monitoring.

### 3.3 Artificial intelligence in skin diagnosis

**Figure 2:**
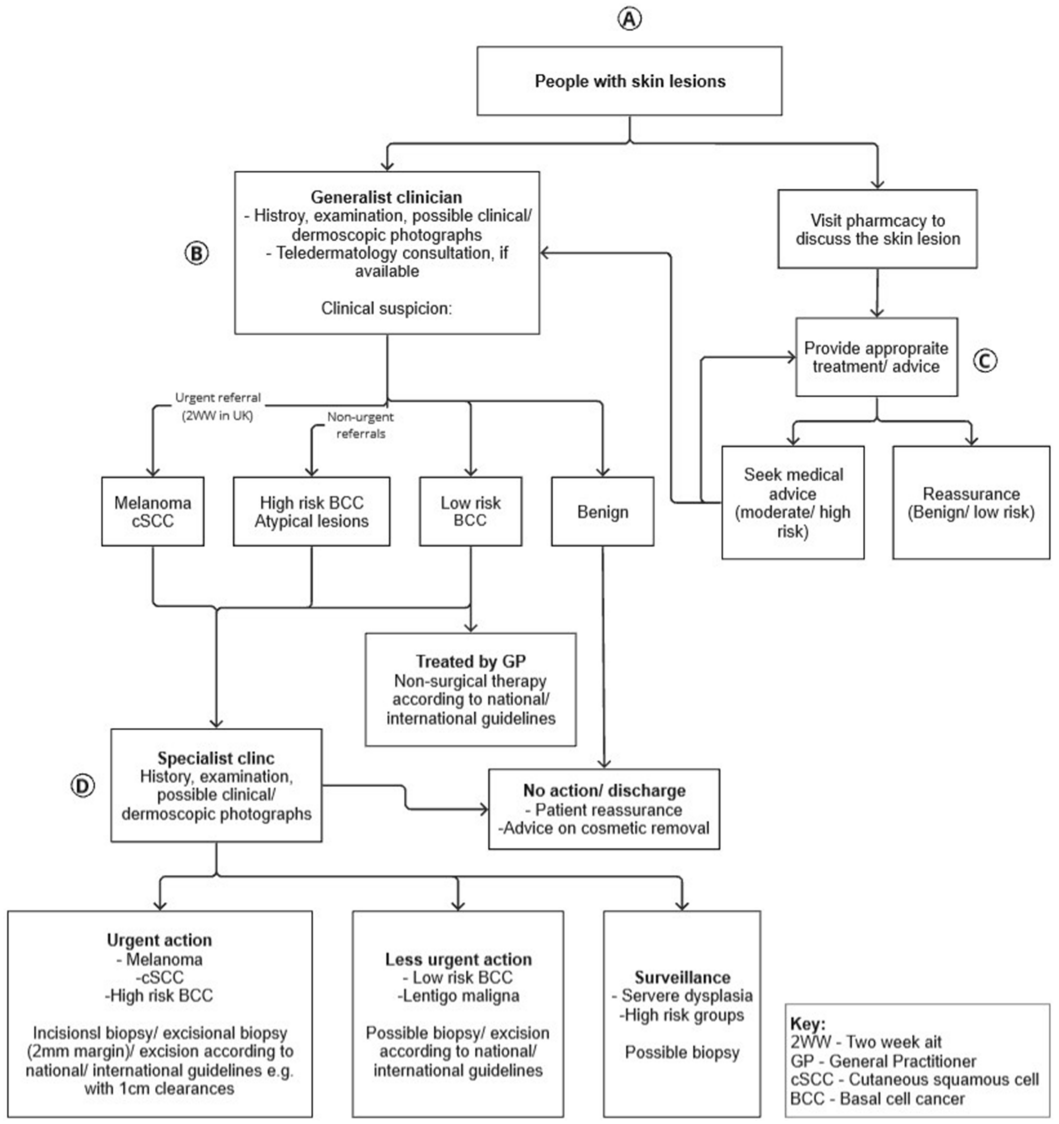
Clinical pathway of skin diagnosis within the UK (Adapted from Chuchu et al., 2018).

The integration of AI in dermatological settings has been investigated on multiple occasions and has proven to achieve the desired results in identifying skin conditions at varying levels (Nasr-Esfahani et al., 2016; Brinker et al., 2019). Considering the limited number of Dermatologists available, within the UK and globally (Eedy, 2015), it would benefit patients, GPs, and Dermatologists for AI to be successfully integrated into the clinical pathway. The current clinical pathway of checking the health of the skin and diagnosing possible conditions, within the UK, is shown in Figure 2. This flowchart has been adapted from the figure presented by Chuchu et al. (2018), illustrating the clinical pathway for skin lesions. The revised version incorporates the UK Government’s guidelines on promoting the Pharmacy First Scheme (Department of Health and Social Care, 2024), which aims to alleviate the burden on GPs by encouraging patients to seek advice or treatment at a pharmacy as an initial step first, or they may choose to consult a GP directly. At the primary care level, skin concerns are categorized as melanoma, high risk, low risk, or benign. High-risk cases and melanoma are referred to Dermatologists, while low or benign cases are treated by GPs, and if no concern is confirmed, patients are discharged. AI integration can occur at various points in the clinical process (Points A, B, C, and D in Figure 2). An AI skin recognition tool at these decision points may assist in diagnosing skin concerns, collecting relevant images and descriptions, and expanding data sets that serve to improve future diagnostic accuracy. Implementing AI at these points could potentially alleviate the workload for primary care providers, whilst providing better outcomes for patients.

AI success consists of these factors:

- Sensitivity: This assesses a model’s ability to predict true positive values of each available category (Mitrani, 2019).
- Specificity: This evaluates a model’s ability to predict true negative values (Mitrani, 2019).
- Area under the receiver operating characteristic curve (AUROC): This is used to measure accuracy on classification tasks, the closer the receiver operating characteristic curve is to the upper left corner of the graph, the higher the accuracy of the test as the upper left corner is where the sensitivity = 1 and the false positive rate = 0 (specificity = 1).
- Receiver operating characteristic (ROC): This is used to evaluate the overall diagnostic performance of a test and to compare the performance of two or more tests (Nahm, 2022). The ideal ROC curve has an AUC = 1.0. However, when the coordinates of the x-axis (1 – specificity) and the y-axis correspond to 1: 1, a graph is drawn on the 45° diagonal (y = x) of the ROC curve (AUC = 0.5). An AUC greater than 0.5 is essential for any diagnostic technique to be meaningful, and it is often required to exceed 0.8 to be considered acceptable (Nahm, 2022).

There are several factors to take into consideration during the development of AI for dermatological use and the impact they can have on its outcome. Overfitting is a significant challenge in supervised machine learning, where models exhibit high accuracy on training data but perform poorly on new data (Aggarwal, 2019). This can be problematic in skin lesion diagnostics due to the variability in data such as, size of skin lesions and variation in the angle images are taken (Aggarwal, 2019). To mitigate overfitting, steps such as data augmentation which help increase diversity and number of images, are taken (Khosla and Saini, 2020).

Data augmentation is the practice of artificially modifying images to account for a variability that exists in image taking (Aggarwal, 2019) and helps to expand training sets. This may be beneficial when limited images of skin conditions are available (Aggarwal, 2019; Chlap et al., 2021; Wen et al., 2022). Supervised machine learning typically relies on substantial amounts of training data to reduce the risk of overfitting; however obtaining well-annotated medical data is challenging, expensive and time-consuming, making data augmentation valuable in such situations. Chlap et al. (2021) categorize data augmentation into three main types:

- Basic augmentation (involving geometric transformations, cropping, occlusion, intensity operations, noise injection, filtering, and combinations)
- Deformable augmentation (utilising random displacement, spline interpolation, deformable image registration, and statistical shape models)
- Deep learning augmentation techniques (including Generative Adversarial Networks (GAN)-based augmentation methods).

Studies (Perez et al., 2018; Abayomi-Alli et al., 2021; Rezk et al., 2022; Saeed et al., 2023) highlight the positive impact of using data augmentation techniques to expand training sets on skin conditions and classification models, including increasing the number of images for POC, which is already very sparse. Although augmentation enhances data diversity, it introduces the risk of generating synthetic patterns that may not accurately represent real data, potentially affecting the model’s performance.

Image selection is a fundamental aspect of AI development for skin diagnosis (Aggarwal, 2019; Brinker et al., 2019; Hogarty et al., 2019). Excluding inadequate low-quality images is essential to maintain a high level of sensitivity and specificity, consequently limiting the amount of usable training data. Low image quality refers to images affected by low resolution, presence of noise or small dynamic range where detail in an image may be lost due to dark or bright areas (Koziarski and Cyganek, 2018). Factors including hair, background skin issues, sun damage, rulers, blurry images, or dark corners of lenses contribute to poor image quality, causing confusion and miscalculation in results (Winkler et al., 2019; Liopyris et al., 2022). Ribeiro et al. (2016) conducted a study looking at AI distinguishing between photos of wolves and huskies. Results indicated that the AI predominantly relied on the entire image to differentiate between a wolve and huskie. Images which contained a light background or snow at the bottom were identified as wolves, if not they were identified as huskies, this is mainly due to images of wolves being taken in the snow. This is an example of overestimating the validity of AI models accuracy and would be problematic, especially for use within healthcare. In the application of AI to skin diagnosis, if a program is familiar with seeing melanoma on Caucasian skin, it may struggle considerably to identify the same on POC.

The study of Nasr-Esfahani et al. (2016) was one of the first to introduce AI into Dermatology; it was used to detect melanoma and benign cases using convolutional neural networks (CNN). CNN refers to a type of neural network where layers apply filters for specific features to areas within an image (Du-Harpur et al., 2020). The dataset for this study comprised of original images and augmented images subjected to cropping, scaled, and rotated and produced promising specificity and sensitivity results (Nasr-Esfahani et al., 2016). The success of the AI being able to distinguish melanoma from benign cases heavily relied on dataset illumination corrections which increased its ability to differentiate between the two conditions.

Brinker et al. (2019) investigated the performance of CNN-based classification of clinical images compared to dermatologists in sensitivity, specificity, and ROC. Dermatologists collectively achieved a mean sensitivity and specificity of 89% and 64%, respectively. In comparison, the CNN demonstrated a mean specificity of 68% and achieved the same sensitivity levels as the dermatologists (Brinker et al., 2019). Similar results are reported in a study by Han et al. (2020): clinicians’ results indicated a sensitivity and specificity of 70% and 96%, respectively, while the CNN achieved 63% and 90%, respectively. Comparable outcomes are presented in Philips et al. (2020) with the AI program achieving 85% for both sensitivity and specificity and dermatologists achieving 87%, and 81%, respectively. The studies highlight promising AI performance and show good prospects of AI integration within dermatological workflows for skin diagnostics. Despite this, each study’s drawback consists of the underrepresentation of POC in its dataset affecting the generalisability of results.

There is a growing body of literature that acknowledges the gravity of POC underrepresentation in AI training datasets. Jinnai et al. (2020) used images of only Black and Brown pigmented skin lesions on a faster region-based convolutional neural network (FRCNN) program. This produced a specificity and sensitivity of 94% and 83%, while board certified Dermatologists produced results of 86% for both sensitivity and specificity (Jinnai et al., 2020). Similar results are seen in Chen et al. (2016) study using images of different ethnicities to assess AI performance in identifying melanoma; sensitivity, and specificity results of 90% and 91% were reported. Liu et al. (2020) study for Google Health produced results of ‘top-1 accuracy’ of 71% and ‘top-1 sensitivity’ of 58% when diagnosing a range of contrasting skin conditions across different skin tones varying from FST I – V. Furthermore, Liu and Primiero (2023) systematic review presented evidence of accurate AI programs for POC within multiple studies showing accuracy levels from 70% to almost 100%.

Despite the observed high levels of accuracy reported in these studies, a comprehensive analysis of the dataset used shows little to no representation of POC data. Liu et al. (2020) study had 2.7% of participants with FST V and 0% of participants with FST VI. Chen et al. (2016) study had a range of ethnic participants but were not in a balanced ratio to Caucasian participants (American Indian or Alaska Native 2%, Asian or Pacific Islander 13.9%, Black or African American 4.3%, White, or Caucasian 30%). Jinnai et al. (2020) study did not provide a breakdown in the number of Brown and Black participants from each FST group, which is key as a limited number of FST VI and a higher number of IV will affect its validity. Additionally, Liu and Primiero (2023) systematic review predominantly consisted of papers with participants of East Asian origin with some studies containing only 10% of participants with FST type IV–VI. Schakermann et al. (2024) study developed the Health Equity Assessment of machine Learning (HEAL) framework to assess the performance of health AI in a case study. While Schakermann et al. (2024) case was carefully sampled to create a balance in demographics, there was still a poor representation of FST V-VI and American Indian/Alaska Natives. These studies’ results are skewed due to poor representation of POC affecting the results generalisability or show the struggle in trying to work with balanced data sets due to limited resources.

Aggarwal (2019) study proves AIs ability to correctly diagnose melanoma through CNN programs. Augmentation of data was carried out by artificially darkening light skin toned images to input into the program. Results produced higher sensitivity (0.82) and specificity (0.76) rates for darker skin images compared to lighter skin tones (0.63 and 0.60). However, the ‘darkening’ of the images was only able to create data belonging to FST II, still excluding FST III – VI groups. This is a result of wanting to preserve the characteristics of the skin lesion on the original light skin toned images. Despite the potential misinterpretation of the study, it still shows the capability of AI accuracy in melanoma diagnosis when training with minimal inclusive data sets. Similarly, Abhari and Ashok (2023) investigation used data augmentation techniques to increase the POC data set to improve the studies accuracy. However, the study generalized darker skin tones and failed to present information on skin tone categories (such as FST), making it difficult to comprehend the breadth of skin tones explored.

AI powered digital tools for skin diagnosis’s have been made publicly accessible. Skin Image Search, developed by First Derm, was established to increase the availability of expert skin information. The application works by uploading two pictures of a skin lesion (an overview and close-up) to produce a diagnosis. The app has been used globally, in countries such as Sweden, Chile, China, Australia, and Ghana. Zaar et al. (2020) assessed the diagnostic accuracy of Skin Image Search developing interesting insights. The dataset consisted of all skin phototypes but low levels of FST type IV (4.2%), V (0.9%) and VI (1.4%) (type I 16.7%, II 59.5%, III 17.2%) were included. Evaluation results also indicated high and low levels of accuracy across varying skin conditions; and a top-5 accuracy rate of 56.4, and 22.8% accuracy for the most probable diagnosis. The poor accuracy rates, with a high FST I, II, and III and low FST IV, V, and VI test images, suggest that the program needs further refinement and development. Kamulegeya et al. (2023) tested Skin Image Search’s diagnostic performance using predominantly FST VI images extracted from The Medical Concierge Group in Uganda. Data sets were anonymised and filtered to ensure a quality dataset was used. Skin Image Search was able to correctly diagnose 17% of images compared to the 69.9% performance reported from the AI training results. The subpar results could indicate that First Derm was heavily trained on images with FST I and II. FirstDerm has stated in a blog that Skin Image Search has an accuracy rate of 80% (Börve, 2021) with no supporting data for the claim. Such disinformation can increase the problems already caused by the underrepresentation of POC by creating a false sense of security among those who take information at face value, further increasing the health inequality gap.

Some AI tools are under development for skin diagnostics. Google has recognized that consumers conduct 10 billion searches annually related to skin, nail, and hair conditions and is now developing Derm Assis (Bui and Liu, 2021). This program operates by users capturing three images of the skin condition, answering questions about their skin type and the duration of symptoms, and then presenting possible diagnosis to the users. Google emphasises that this tool serves as an ancillary support, providing users with information before deciding on their next steps. Google’s Health study for the development of the deep learning system, revealed a top differential diagnosis in validation with an acceptable accuracy and sensitivity rate when given the option to provide one diagnosis (Liu et al., 2020). When given the chance to provide three diagnoses, accuracy and sensitivity levels were significantly better across all 26 skin conditions (Liu et al., 2020). While there are promising results, Google’s identification of consumer need with the current response of a dermatological level tool, fails in its generalization ability. This is a consequence of using a dataset that is not representative of all ethnic groups; groups with skin tones in categories FST V were represented by 2.7% of participants and 0% for FST VI. This action formulates potential misdiagnoses and biases, especially among ethnic groups.

## 4 Discussion

Health inequalities have been tackled in multiple ways through strategies and digital technological approaches. The NHS Core20PLUS5 strategy presents a targeted approach to reducing health inequalities with a focus on specific communities and groups. The future success of this strategy could also serve as a foundation for tackling inequalities in health globally, considering the impact on population composition that economic and political migration are generating. Other approaches including Healthy.io, NHS Croydon Primary Care Trust Virtual AI Ward, and the USA medical system present the case of successful AI capabilities in addressing health inequalities through ease and appropriate access to medical care, treatment, and results with the condition that it is supervised correctly suggesting that unsupervised AI would not be appropriate, and possibly detrimental, in medical settings.

Achieving success in tackling health inequalities through AI usage in complex areas such as dermatological settings is possible. However, for such success to occur some foundational issues must be resolved first to create the conditions for an effective and rigorous application of AI. The NHS (2022) approach to the expansion of the Health A-Z free public website and Malone Mukwandes’ Mind the Gap initiative (https://www.blackandbrownskin.co.uk/mindthegap) emphasise the limited representation of POC in current data sets, and the possibilities of false-positive reassurance in self-diagnoses when primary care follow ups are not carried out. The inadequate representation of skin tones is commonly seen within research and educational settings as a reoccurring issue (Lester et al., 2019). This is a barrier faced by many researchers and has consistently been a failure in AI development, despite the attempts made through data augmentation. Whilst data augmentation creates the potential to expand the dataset of POC through various techniques, it creates the possibility of generating synthetic patterns that are unrepresentative of the real population. This could be detrimental not only to a particular study’s reliability, but generally to public trust in AI usage in healthcare.

Within dermatology, it is evident that the capability of AI to match or surpass dermatologists’ performance is achievable. Addressing challenges such as overfitting and implementing effective data augmentation is important for the development and accuracy of AI in the diagnosis of skin lesions. Ensuring diversity in image datasets is equally crucial to prevent biases, as highlighted by multiple studies that demonstrated poor diagnostic performance when AI was predominantly trained on lighter skin tones. Some studies claim to include POC in training datasets or in the testing of AI programs, suggesting insightful findings; however, looking specifically at the number of POC data used, it is clear that statistical representation has yet to be achieved. Not only are more patients of color needed within studies, but transparency and clarity from researchers on participant skin tones need to be shared to avoid misleading interpretations. The consistent use of the FST scale throughout clinical studies could be considered a contributing factor to the lack of POC representation. The scale is currently inclusive of non-marginalized and ethnoracial minorities alike (Heldreth et al., 2024), compressing under type IV-VI a plethora of diverse skin tones that are therefore unfairly represented in the scale. This creates poor dermatological learning resources and, consequently, AI studies in dermatology.

Before AI can be used, within clinical studies, for skin diagnostic purposes several interventions need to take place to reduce biases and to show the potential and reliability of AI. This can be achieved in many ways, including:

- An increased database of expert confirmed diagnoses across a variety of skin tones.
- Targeted campaigns for hard-to-reach groups. This will result in higher participation of POC in clinical studies.
- An improvement in learning resources providing accurate and diverse clinical representation of POC through detailed supportive text and images.
- Continuous professional development (CPD) for GPs to create a better understanding of unintentional biases and awareness of skin lesions among POC.
- An appropriate skin color categorization technique, which encapsulates different skin color variations, and can also be used within clinical and educational settings.

Without these interventions in place, the systemic issue of the under representation of POC in AI cannot be solved and will only continue to amplify the disparities and exclusion POC face.

The limitation of this study includes the lack of full details in the reviewed literature about skin tones used for training data, making it difficult to understand if the findings are generalisable. Additionally, it is unclear whether the literature on AI being reviewed used the same AI programming system. For instance, Brinker et al. (2019) and Han et al. (2020) highlight, in their methodology, the use of CNN, while Jinnai et al. (2020) study uses FRCNN, but Abhari and Ashok (2023), Liu et al. (2020) and Philips et al. (2020) AI programming systems are not clarified. The lack of clear parameters can make it harder to compare the performance of different AI approaches. A clinical validation of the findings highlighted in this review could have also been beneficial.

## 5 Conclusion

Evidence demonstrates a notable disadvantage for POC in various aspects of healthcare. This is seen for skin diagnostics within clinical studies at both primary and secondary care levels. These situations result in lower survival rates, poorer quality of life for POC in comparison to Caucasians, and a disproportionate underrepresentation of POC in medical advancements.

Digital technologies, including the integration of AI, in dermatology have shown promise within healthcare, particularly in addressing the scarcity of dermatologists globally and in providing accurate diagnoses of skin conditions when executed efficiently, as shown through the NHS Croydon Primary Care Trust Virtual AI ward (Hakim, 2023). However, challenges have unexpectedly emerged in AI development that require attention and upstream interventions to improve the lack of diverse representation impacting the reliability and generalisability of AI models. This has also inadvertently highlighted ongoing issues faced by POC within healthcare, such as unintentional biases made by healthcare professionals or incorrect diagnoses of skin conditions. While interesting techniques, such as data augmentation, show potential in overcoming problems, such as the number of limited imagery available on POC, they do not address the unintentional biases shown within healthcare and show the need for more care to be placed in ensuring POC are being cared for at the same pace and level as Caucasians.

To ensure technology advancements continue and to prevent the widening of pre-existing racial disparities, the inclusion of POC in studies needs to be a priority and can be achieved through targeted campaigns to include hard to reach participants. A more effective approach to categorising POC to ensure a comprehensive representation of skin tones is also needed. The current use of the FST scale to represent POC fails to encompass the full diversity of human skin tones. Relevant participant data, such as ethnicity and skin tone, also needs to be transparently shared within clinical studies for a clearer understanding on whether studies are truly generalisable.

Digital tools including Healthy.io and the NHS Croydon Primary Care Trust Virtual AI Ward are successful in their execution, which could be due to the user-centred approach applied. Many studies have taken a technical approach to address skin diagnosis among POC through AI. Comparatively fewer studies have adopted a user-centred approach throughout their development process. Whilst AI-augmented skin diagnosis is technically promising, caution, additional research, measures and regulations are needed. The fundamental issue of the lack of balanced data set representation of all skin types and transparency in research is a gap that needs addressing for both traditional clinical diagnosis and AI-assisted diagnostic pathways.

## Author contributions

NK: Writing – original draft, Writing – review & editing. GS: Supervision, Writing – review & editing. FC: Supervision, Writing – review & editing.
